# Safe Zone to Avoid Pneumothorax in a CT-Guided Lung Biopsy

**DOI:** 10.3390/jcm12030749

**Published:** 2023-01-17

**Authors:** Nour Maalouf, Mazen Abou Mrad, Daniela Lavric, Lora Vasileva, Andreas H. Mahnken, Jonas Apitzsch

**Affiliations:** 1 Department of Radiology and Nuclear Medicine, Helios Hospital Pforzheim, 75175 Pforzheim, Germany; 2 Department of Anesthesiology, Helios Hospital Pforzheim, 75175 Pforzheim, Germany; 3 Department of Diagnostic and Interventional Radiology, University Hospital of Marburg, 35043 Marburg, Germany

**Keywords:** pneumothorax, chest CT, lung biopsy, biopsy needle, needle–pleura angle

## Abstract

Pneumothorax is one of the most frequent complications of computed tomography (CT)-guided lung biopsies. We aim to identify the safe zone of the needle–pleura angle during a CT-guided lung biopsy. Fifty-two patients underwent CT-guided lung biopsies between January 2020 and September 2022 (27 males, 25 females, median age 70 years). Right and left needle angles were measured and correlated to the incidence of pneumothorax. The minimum delta (δ_min_) was calculated as the absolute value of the difference between a 90° angle and the right and left angles. *t*-test *p*-values for δ_min_ were conducted. We recorded 29 patients with pneumothorax, including intraprocedural and transient, postprocedural with minimal symptoms, or postprocedural requiring a chest tube insertion. Thirty-two patients had a δ_min_ ≥ 10°, while 20 had a δ_min_ < 10°. Of the patients with a δ_min_ < 10°, 30% experienced pneumothorax compared to 71.8% in patients with δ_min_ ≥ 10° (*p* = 0.0023). The study results show that as the needle’s angle deviates from the perpendicular, with an absolute value of more than 10°, the likelihood of pneumothorax increases significantly. A needle–pleura angle between 80° and 100° gives the operator a safe zone to reduce the risk of pneumothorax.

## 1. Introduction

Computed tomography (CT)-guided lung biopsy is a standard and acceptable procedure for histopathological confirmation and diagnosis of different lung nodules [1]. The method has a fairly low overall complication rate and a low risk of major complications. However, pneumothorax is still the most common complication of CT-guided lung biopsy at 25%, with an incidence rate ranging between 8% and 61% [2,3]. In a recent retrospective study that included 325 patients, 11.2% of CT-guided lung biopsies were interrupted and discontinued by pneumothorax [4].

Preventing pneumothorax is essential to avoid unnecessary pain and shortness of breath, to reduce the number of interrupted procedures, and subsequently to increase diagnostic accuracy and decrease the days of postprocedural hospitalization while minimizing costs for patients [5].

Several recent studies have highlighted the significance of the needle’s angle in the pleura and the relationship between the right angle and the incidence of pneumothorax [4,6]. This paper aims to correlate the frequency and severity of pneumothorax to different needle–pleura angles, allowing the operator to guide the needle through a safe zone with a lower risk of pneumothorax.

## 2. Materials and Methods

During the period from January 2020 to September 2022, 52 patients (27 males, 25 females; median age: 70 years; age range: 51–88 years) underwent CT-guided biopsies of lung nodules. The CT-guided lung biopsies were performed using an 18G semi-automated Tru-Cut needle (Möller Medical GmbH, Fulda, Germany) and a 17G trocar (Möller Medical GmbH, Fulda, Germany) in a supine or prone position. Right and left angles of the needle to the pleura were measured and correlated to the presence of pneumothorax as an intra-procedural or early post-procedural complication. We calculated a minimum delta as the absolute value of the difference between a 90° angle (perpendicular to the pleura) and the right and left angles obtained. Throughout the procedure, the entire needle was in the plane at a slice thickness of 2.4 mm, which eliminated needle deviation on the *z*-axis. *t*-test *p*-value for the minimum delta was calculated. All patients provided informed consent more than 24 h prior to the intervention. Ethical approval was given by the local ethics committee (F-2021-038).

### 2.1. Inclusion and Exclusion Criteria

This observational study included 52 patients who underwent CT-guided lung biopsies for histological diagnosis of nodules. Exclusion criteria were as follows: a lesion with a diameter <4 mm, International Normalized Ratio (INR) of >1.5, and incapacity to follow instructions or refusal to comply with the procedure.

### 2.2. Semi-Automated Tru-Cut 18G Needle

The 18G biopsy needle has a semi-automated Tru-Cut design with a central sharp stylet surrounded by a hollow cylindrical sheath. The Tru-Cut design needle has become a standard tool used in diagnostic lung biopsies, as it is characterized by superior convenience [7].

### 2.3. 17G Trocar

The 17G trocar (Möller Medical GmbH) facilitates multiple biopsies of the same intrapulmonary lesion with only one pleural passage, as it acts as a guide for the 18G needle.

### 2.4. Biopsy Protocol

In accordance with the pre-intervention images, a selective CT scan of the region of interest was obtained at the end-expiration breath-hold. A 128-slice Siemens Somatom Definition Edge CT (Forchheim, Germany) was used with the following protocol: a 20 mA current at 120 kV, with a 2 mm slice thickness at 1 mm increments. We planned the intervention, disinfected and prepared the puncture site, administered local anesthesia (Mepivacaine 1%), and created a small incision in the skin. We proceeded to place the coaxial needle within the margin of the lesion and introduced the 18G needle through the trocar to take a sample for biopsy. We fixed the obtained samples in formaldehyde and sent them for histopathological analysis. After removing the biopsy needle and closing the puncture site with a sterile patch, a low-dose non-contrast control scan of the chest was performed in end-expiration to assess for intervention-related complications. No other techniques such as blood patching was used on any of the patients. There was never more than a single pleural passage associated with the trocar needle.

### 2.5. Measurement Yield

We digitally measured the angle while the needle was fully detectable in the axial plane. We measured the right and left angles with respect to the patient’s pleura using the picture archiving and communication system (PACS) CHILI (CHILI GmbH, Dossenheim, Germany) for angle measurements.

### 2.6. Statistical Analysis

The right and left angles were recorded following assessment by the interventional radiologist and retrospectively evaluated. We conducted a nominal two-sided *t*-test for the continuous variables and statistical analysis using Excel (version 14.0.4760.1000) (Microsoft Corporation, Redmond, WA, USA) and Gigacalculator [8]. A *p*-value < 0.05 was considered statistically significant.

## 3. Results

**Figure 1:**
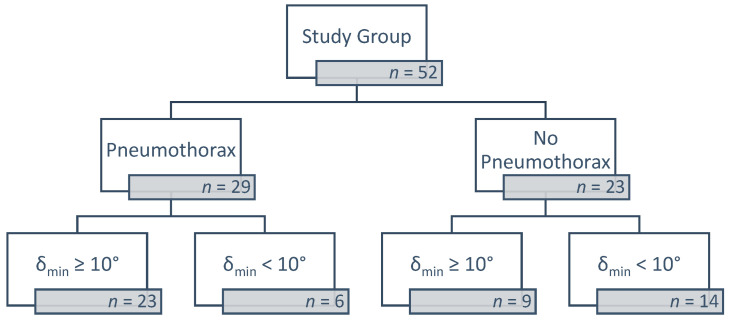
Flow chart of the patient classification process.

Fifty-two patients underwent CT-guided lung biopsies conducted by the same operator with more than 15 years of experience in interventional radiology. We documented the needle–pleura angles for all patients treated from January 2020 to September 2022. We calculated a minimum delta (δ_min_) as the absolute value of the difference between a 90° angle (perpendicular to the pleura) and the right and left angles obtained. Of the 52 patients, 29 experienced pneumothorax, ranging from intra-procedural and transient to post-procedural with minimal symptoms and post-procedural with pronounced symptoms requiring a chest tube insertion. Of the 52 patients, 32 had a δ_min_ ≥ 10°, while 20 had a δ_min_ < 10° (Figure 1). Of the patients with a δ_min_ < 10°, 30% experienced pneumothorax compared to 71.8% in patients with δ_min_ ≥ 10° (*p* = 0.0023).

**Figure 2:**
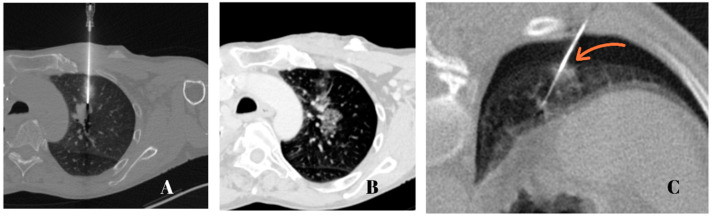
Axial CT images of the lungs at 75 mA, 120 kV, and a 2 mm slice thickness with a 1 mm increment: (**A**) A pulmonary mass is seen in the left upper lobe as the needle is entering the pleura at a 90° angle; (**B**) Post-intervention control scan showing a slight needle tract hemorrhage with no evidence of pneumothorax; (**C**) An intraprocedural pneumothorax as the needle enters the pleura at a very steep angle to target a pulmonary nodule (orange arrow) in the right lower lobe.

All biopsies were planned to avoid important vessels, bullae, fissures, and ribs. During the study, only one biopsy (1/52) had to be discontinued due to a clinically significant pneumothorax that required further interventions. Otherwise, the majority of patients who developed pneumothorax did so after the biopsy was taken. Figure 2A–C demonstrate a CT-guided lung biopsy at two different angle approaches with opposite outcomes.

**Figure 3:**
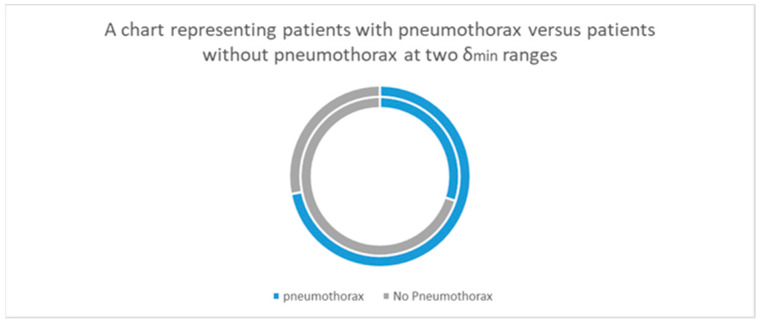
A chart representing patients with pneumothorax versus those without pneumothorax at two δ_min_ ranges. The inner circle represents δ_min_ < 10° and the outer circle represents δ_min_ ≥ 10°.

As displayed in Figure 3, the risk of pneumothorax was significantly higher in patients with a needle–pleura angle less than 80° or more than 100°. However, the inner circle of the chart highlights the reduced incidence of pneumothorax when δ_min_ was lower than 10°.

**Figure 4:**
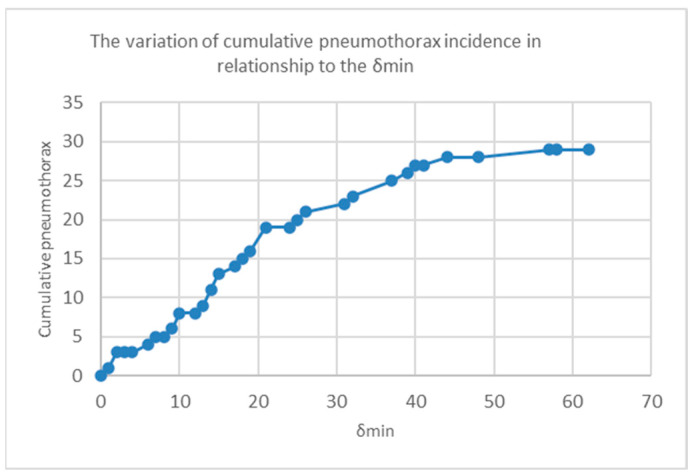
The variation in cumulative pneumothorax incidence in relation to the δ_min_.

Figure 4 shows the variation in the cumulative incidence of pneumothorax in relation to δ_min_. As the needle–pleura angle deviated away from δ_min_ of 10°, we observed a significant increase in the cumulative incidence of pneumothorax.

**Figure 5:**
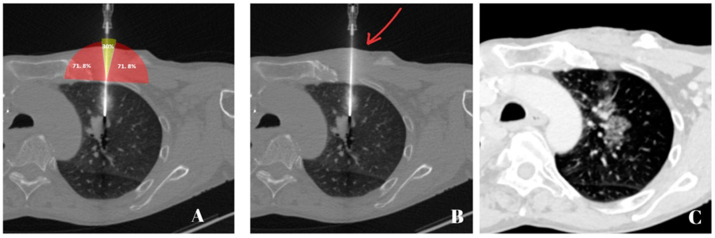
Different axial CT images of the same patient at 75 mA, 120 kV, and a 2 mm slice thickness with a 1 mm increment: (**A**) A safe zone scheme was placed on the needle before it entered the pleura. The yellow triangle depicts δ_min_ < 10° and the red triangles depict δ_min_ ≥ 10° with a significantly higher incidence of pneumothorax (71.8% vs. 30%); (**B**) The same image as presented in (**A**) but without the safe zone scheme. The red arrow points to the needle that has entered the lung at a 90° angle to the pleura, aiming for the presented pulmonary mass in the upper left lobe; (**C**) Post-intervention control scan showing no evidence of pneumothorax.

Of the patients who had a δ_min_ < 10°, 30% experienced pneumothorax compared to 71.8% of the patients who had a δ_min_ ≥ 10° (*p* = 0.0023), as presented in Figure 5. Only 1 of 20 patients (5%) who had a δ_min_ < 10° needed a chest tube, compared to 8 of 32 patients (25%) who had a δ_min_ ≥ 10° (*p* = 0.0017).

## 4. Discussion

This study determined the incidence of pneumothorax for different biopsy needle angles in the pleura. Our retrospective study included 52 patients who underwent CT-guided lung biopsies. We evaluated the incidence and severity of pneumothorax at different needle–pleura angles while using the same needle gauge and brand throughout the study. All procedures were performed by the same operator. Various previous studies have evaluated the importance of the needle–pleura angle and its significance in the incidence of pneumothorax [3,6,9,10,11]. A multivariate analysis by Hiraki et al. involving 1098 patients found that a needle trajectory angle of <45° (*p* = 0.014) was a significant risk factor for pneumothorax [9]. These results are in accordance with the findings of Ko et al. and Saji et al., who showed that needle–pleura angles of less than 80° yield a higher risk of pneumothorax development [10,11]. The significance of the needle–pleura angle was also assessed by Sheikh et al. in 2019, who studied CT-guided lung biopsies of 208 patients and noticed a relationship between the needle–pleura angle and the incidence of pneumothorax (*p* = 0.02) [12]. The author also highlighted the lowest rate of pneumothorax to be 14.8% between 80° and 90° [12]. This is consistent with our results that show a significant increase in the occurrence of pneumothorax as the needle–pleura angle deviated 10° or more from an acute angle (30% vs. 71.8% *p* = 0.0023). Since patients with a needle–pleura angle of less than 80° or more than 100° have a significantly higher chance of pneumothorax, we recommend the use of evidence-based mitigation techniques such as the rapid needle-out patient-rollover technique [13]. However, a study by Chami et al. involving 163 patients showed no significant relationship between the needle–pleura trajectory angle and the occurrence of pneumothorax [14].

Of the 52 patients, 29 experienced pneumothorax during our study. This inflated number is largely due to the instances of transient, small, and intra-operative pneumothorax that we included while classifying the patients, even though they were asymptomatic. Eleven patients required chest tube placement (21.1%). This high incidence may be explained by the fact that 21.1% (11/52) of our study group was aged 80 years and above, with two patients being 88 years old. Previous studies have suggested that the chest tube placement rate is between 0% and 53% among patients with pneumothorax [10,14,15,16,17]. Although Chami et al. found no correlation between pneumothorax and older age, with the mean age of patients in their study being 61 years (SD = 13.7) compared to our patient pool with a mean age of 69 years (SD = 10), Kuban et al. (2015) did find a statistical significance between older age and the rate of pneumothorax in a study including 3917 patients [14,18]. Some studies have shown that the 18G needle is the needle of choice for CT-guided lung biopsies because it causes minimal tissue damage, and using a larger needle increases the prevalence of pneumothorax and chest tube placement [18,19].

Our study was limited to a relatively small number of patients, as it was a single-center observational study. All the biopsies were carried out by a single experienced interventional radiologist, which might have restricted our results, as the operator is considered the third risk factor in the incidence of pneumothorax; therefore, including more operators with different years of experience might lead to different results [15]. The included CT measurements were recorded and assessed objectively, thus reducing bias and rendering the results generalizable, as the incidence of pneumothorax and chest tube placement in this study was analogous to those reported in previous studies with a larger patient pool. This study is particularly important, as it sheds light on how minute changes in the needle–pleura angle can reduce the incidence of pneumothorax. A future study with a larger patient pool and inclusive multivariate analysis may be useful in enabling new specific guidelines for CT-guided lung biopsies.

## 5. Conclusions

In conclusion, the more the needle angle deviates from the perpendicular with an absolute value of more than 10°, the greater the likelihood of pneumothorax occurrence. In other words, this gives the operator a triangular safe zone between 80° and 100° to acquire a pulmonary nodule sample while keeping the risk of pneumothorax low.

## Data Availability

The data presented in this study are openly available in FigShare at Maalouf, Nour; Apitzsch, Jonas (2022): Safe zone. figshare. Dataset: https://doi.org/10.6084/m9.figshare.21201764.v1, accessed on 4 September 2022.
